# Marital status and its effect on lung cancer survival

**DOI:** 10.1186/2193-1801-2-504

**Published:** 2013-10-03

**Authors:** Stacey L Tannenbaum, Wei Zhao, Tulay Koru-Sengul, Feng Miao, David Lee, Margaret M Byrne

**Affiliations:** Sylvester Comprehensive Cancer Center, Miller School of Medicine, University of Miami, 1120 NW 14th Street, Miami, FL 33136 USA; Department of Public Health Sciences, Miller School of Medicine, University of Miami, Miami, FL USA; Department of Surgery, Miller School of Medicine, University of Miami, Miami, FL USA

**Keywords:** Lung cancer, Marital status, Outcomes, Florida population-based cancer registry, Support system

## Abstract

**Purpose:**

The purpose of this study was to determine if marital status, including specific types of single status categories, is associated with length of survival in lung cancer patients.

**Methods:**

Data from the 1996–2007 Florida Cancer Data System were linked with Agency for Health Care Administration data and U.S. Census data. Patients with both small cell and non-small cell lung cancer were identified (n = 161,228). Marital status was characterized by married, widowed, separated/divorced, and never married. We compared median survival time and 1, 3, and 5-year post diagnosis survival rates.

**Results:**

Overall, 54.6% were married, 19.1% were widowed, 13.5% were separated/divorced, and 12.7% had never married. Median survival in months was longest for married (9.9) and widowed (7.7) patients, and shortest for never married (4.9) and separated/divorced (4.1) patients. Five-year survival rates were 14.2% for married, 10.7% for widowed, 8.9% for separated/divorced, and 8.4% for never married. In univariate Cox regression, marital status was a significant predictor of better survival for married (HR = 0.70; p < 0.001) and widowed (HR = 0.81; p < 0.001) patients compared with never married patients, but worse for separated/divorced patients (HR = 1.03; p = 0.003). Multivariate models demonstrated sustained survival benefits for married (HR = 0.86; p < 0.001) and widowed (HR = 0.88; p < 0.001) patients, and detriments for separated/divorced patients (HR = 1.05; p < 0.001) after adjusting for extensive confounders including demographics; tumor stage, grade, and morphology; comorbidities; treatment; and smoking status.

**Conclusions:**

Our study demonstrated that married or widowed lung cancer patients have better survival compared to patients who were never married or separated/divorced. Research to understand the mechanism of this effect, and how the beneficial effect can be extended to those who have never married or have had the marital relationship severed through divorce or separation is needed.

## Introduction

Lung cancer is the second most common cancer in the U.S. but is responsible for the greatest number of deaths from cancer (American Cancer Society 2013). In 2013, it is estimated that there will be 246,210 new cases and 163,890 deaths ascribed to lung cancer (Siegel et al. 2013). Estimations for 2013 are that 14% of all incident cancers will be from lung cancer, with 28% of all cancer specific deaths in men and 26% of all deaths in women being attributable to lung cancer (American Cancer Society 2013). Despite advances in chemotherapy and radiotherapy, the 5 year survival rate for all stages combined is estimated to be approximately only 16% (Siegel et al. 2013).

Because of these dismal statistics, it is important to explore all factors that might positively affect survival and mortality outcomes. Recent and growing literature suggests that psychological factors and the presence or absence of social support may be an important factor influencing the course of cancer (Ikeda et al. 2013; Pinquart & Duberstein 2010; Cassileth et al. 1988; Rendall et al. 2011); this has been shown to be especially strong for breast cancer (Falagas et al. 2007; Nausheen et al. 2009). There have been mixed results in the literature regarding the specific association of lung cancer survival and marital status. One study showed that marital status is an independent factor for predicting overall survival in both men and women (Kravdal & Syse 2011). However another found that marriage was not significantly predictive of survival (Siddiqui et al. 2010), and others found some benefits to marriage for men (Saito-Nakaya et al. 2008). The purpose of this study was to assess, using a large comprehensive population-based dataset, whether marital status is an independent predictor of lung cancer survival.

## Methods

### Data

Data from two databases (1996–2007) were linked via patient ID number to form the base dataset for this study: The Florida Cancer Data System (FCDS) data and Florida’s Agency for Health Care Administration (AHCA) dataset. The matches were confirmed with the patient’s date of birth and gender. In addition, patients’ residency was used to approximate patient level socioeconomic status (SES). From the U.S. Census, we obtained tract-level information on the percentage of households in a tract with income below the federal poverty line. Each tract was categorized as: lowest (≥20%), middle-low (≥10 and <20%), middle high (≥5 and < 10%), and highest (<5%) SES based on percentage of households in the tract living in poverty. Individuals living in each tract were assigned that tract’s SES level.

Diagnoses and procedure codes on all patients with lung cancer treated at Florida in- and out-patient hospitals and free-standing surgical and radiological treatment centers were obtained from the AHCA database (Agency for Healthcare Administration 2012).

The FCDS is a population-based registry mandated by law to report all cases of cancer in the state of Florida, with the exception of those diagnosed and treated by the Veterans Affairs. Approximately 95% of all incident cases of cancer are captured. Our sample is representative of the population of lung cancer patients in Florida. As we were only interested in lung cancer, we included only those cases coded as lung cancer in the registry. From FCDS data, we captured incident cases of lung cancer, stage of disease at diagnosis and other disease characteristics, medical history, patient demographics, and methods of treatment (Florida Cancer Data System 2012).

Although we used only lung cancer cases in Florida, using FCDS data has several advantages over the main alternative, which is SEER data. First, we had the ability to link the registry data to an administrative database, AHCA data, which enabled us to enrich our control variables with information on all diagnoses and procedures. Being able to account for all comorbidities is a major strength of the study. Second, although SEER-Medicare linked data is available and would have allowed for analyses that include diagnoses, this would largely be restricted to patients 65 years and older. Our population, on the other hand, covers an age range from 18 to 110 years old. As the development of cancer in those living below the poverty line, among tobacco users, and among certain minorities commonly occurs at a younger age, a restriction to 65 years and older with the SEER-Medicare data would be much more limiting.

### Variables

Overall survival, our primary endpoint, was defined as time from diagnosis to date of death or last follow-up date.

FCDS data was used to determine date of death. If FCDS did not have a date of death, FCDS and AHCA data were compared to obtain the latest date of contact. Patients without a date of death were considered to have censored data and could either be alive, or be dead and have been lost to follow up in the FCDS through moving out of the state or some other means. Our main predictor of interest was marital status which was categorized as married, widowed, separated/divorced, or never married. Following the methodology of other studies (e.g., 9,14-16), we combined separated and divorced patients into one category. In Florida, legal separation is not necessary prior to getting divorced but there are provisions of the law whereby separated partners receive the same alimony and child support payments as do divorced partners. In addition, getting divorced in Florida is easy and quick, and so divorce may be as attractive an option as separation in some cases. Therefore, those in the separated and divorced categories are likely to be more similar to each other than to other categories. Also, as the total number in the separated category was small (3.2% of the total sample), it was not feasible to analyze them separately.

Other factors used as covariates in the regression models were added in a sequential-block stepwise fashion. Demographic characteristics included race (White, Black, Other), ethnicity (Hispanic, non-Hispanic), socioeconomic status (SES; lowest [≥20% of the tract living below the federal poverty line], middle-low [≥10% and <20%], middle-high [≥5% and <10%] and, highest [<5%]), gender, primary payer at diagnosis (private, Medicaid, Medicare, Defense/Military/Veteran, Indian Health System, uninsured, other), smoking status (never, history, current), treatment facility characteristics (teaching, non-teaching; high volume, low volume), and geographic location (rural, urban). Clinico-pathological characteristics were tumor grade (undifferentiated, poorly-differentiated, moderately-differentiated, well-differentiated, other), tumor SEER summary stage (localized, regional direct extension with or without lymph nodes, regional lymph nodes only, distant), lymph node status (positive, negative), type of treatments (chemotherapy [yes/no], radiation [yes/no], surgery [yes/no]), and type of cancer (non-small cell, small cell). The final block of covariates added to the full model was the 31 Elixhauser comorbid conditions (yes/no) based on ICD-9 codes in the AHCA database.

### Population

Our sample included all patients ≥18 years diagnosed with lung cancer (1996–2007) in the state of Florida (n = 179,630). We continued to follow this cohort for a 3-year period through 2010 to determine whether patients had died in this follow-up period. Non-Florida residents and patients with missing values for marital status, race, ethnicity, or SES were excluded (n = 18,402), resulting in a total sample size of 161,228.

### Statistical analyses

Chi-square tests for contingency tables were used to examine the association of categorical variables. Overall median survival time and 1-, 3-, and 5-year survival rates were estimated by the Kaplan-Meier method. Log-rank tests were used to compare the survival rates by marital status. Univariate and multivariate Cox proportional hazards regression models were used to obtain unadjusted and adjusted hazard ratios (HR) and 95% confidence intervals (95% CI). Models were adjusted by adding blocks of variables sequentially whereby model 1 was univariate with marital status as the sole explanatory variable; model 2 was multivariate adjusted for race, ethnicity, and SES; model 3 was model 2 plus all remaining demographic characteristics; model 4 was model 3 plus all clinico-pathologic characteristics; and model 5, the full model, was model 4 plus all comorbidities. Because the effect of marital status has been shown to vary by gender, we considered stratification by gender for our analyses. However, when testing for interactions between gender and marital status in the multivariate Cox regressions, no interactions were found. Therefore, gender was included as an independent predictor of survival in the models.

Patients treated in the same hospital or facility share some unmeasured characteristics that may affect clinical outcomes and therefore cannot be considered as independent observations. Thus, robust standard errors to adjust for clustering of patients within medical facilities were calculated for all models. The type-I error rate was set at 5%. The SAS v9.3 (SAS Institute Inc., Cary, NC) was used to perform all analyses. This project was approved by the University of Miami Institutional Review Board.

## Results

### Patient demographics and clinical variables

Sociodemographic and clinico-pathologic characteristics of the sample are reported in Tables 1 and 2. Overall, 54.6% of the patients were married, 19.1% widowed, 13.5% separated/divorced, and 12.7% never married. The majority of the patients were male (55.7%), White (92.5%), non-Hispanic (93.9%), and in the middle-high and highest SES category (54.8%). Widowed patients were the oldest (median age 7.62 years, range 23–105) followed by married (69 years, range 20–104) and never married (65 years, range 18–102). More married and widowed patients received Medicare insurance (58.4 and 76.3%, respectively) than did never married (35.8%) or separated/divorced patients (34.6%). Overall, 84.5% of the patients had more than 4 comorbidities; a larger proportion of married (87.6%) and widowed (88.2%) had more than 4 comorbidities than did never married (76.3%) or separated/divorced (74.2%). More married and widowed patients were diagnosed at the localized stage (18.3% and 18.2%, respectively) than separated/divorced (11.8%) and never married (11.3%). The proportion of patients with the more treatable non-small cell lung cancer was higher in married (64.5%) and widowed (60.2%) compared with separated/divorced (47.1%) and never married (51.1%).

**Table 1:** **Demographic characteristics of lung cancer by marital status**

Variable		All patients	Marital status at DX							
				Never married		Separated/Divorced		Widowed		Married
			N	%	N	%	N	%	N	%
All patients	161,228	100.0	20,528	100.0	21,789	100.0	30,866	100.0	88,045	100.0
Marital status at DX										
Never married	20,528	12.7	20,528	100.0	-	-	-	-	-	-
Separated/Divorced	21,789	13.5	-	-	21,789	100.0	-	-	-	-
Widowed	30,866	19.1	-	-	-	-	30,866	100.0	-	-
Married	88,045	54.6	-	-	-	-	-	-	88,045	100.0
Race										
White	149,178	92.5	17,163	83.6	19,844	91.1	28,941	93.8	83,230	94.5
Black	10,975	6.8	3,227	15.7	1,826	8.4	1,767	5.7	4,155	4.7
Other	1,075	0.7	138	0.7	119	0.5	158	0.5	660	0.7
Hispanic origin										
Non-Hispanic	151,442	93.9	18,783	91.5	20,442	93.8	29,520	95.6	82,697	93.9
Hispanic	9,786	6.1	1,745	8.5	1,347	6.2	1,346	4.4	5,348	6.1
SES										
Lowest	20,668	12.8	4,674	22.8	3,723	17.1	3,755	12.2	8,516	9.7
Middle-Low	52,264	32.4	6,912	33.7	7,818	35.9	9,999	32.4	27,535	31.3
Middle-High	60,415	37.5	6,453	31.4	7,334	33.7	12,053	39.0	34,575	39.3
Highest	27,881	17.3	2,489	12.1	2,914	13.4	5,059	16.4	17,419	19.8
Vital status										
Alive	21,919	13.6	2,376	11.6	2,332	10.7	3,685	11.9	13,526	15.4
Dead	139,309	86.4	18,152	88.4	19,457	89.3	27,181	88.1	74,519	84.6
FCDS tobacco use										
Never smoke	14,001	8.7	1,409	6.9	1,068	4.9	3,683	11.9	7,841	8.9
History smoke	64,008	39.7	5,247	25.6	5,244	24.1	13,505	43.8	40,012	45.4
Current smoke	54,425	33.8	7,989	38.9	8,031	36.9	9,711	31.5	28,694	32.6
Unknown	28,794	17.9	5,883	28.7	7,446	34.2	3,967	12.9	11,498	13.1
Age at diagnosis										
Mean		69.8		65.2		67.9		76.2		69.0
Std		11.2		12.6		12.1		8.7		10.4
Median		71.0		66.0		68.0		77.0		70.0
Q1		63.0		56.0		59.0		71.0		63.0
Q3		78.0		75.0		76.0		82.0		76.0
Min		18.0		18.0		25.0		23.0		20.0
Max		110.0		102.0		110.0		105.0		104.0
Sex										
Female	71,386	44.3	7,233	35.2	11,256	51.7	22,236	72.0	30,661	34.8
Male	89,842	55.7	13,295	64.8	10,533	48.3	8,630	28.0	57,384	65.2
Insurance status										
Uninsured	5,486	3.4	1,672	8.1	1,222	5.6	426	1.4	2,166	2.5
Private insurance	30,342	18.8	3,539	17.2	3,419	15.7	3,973	12.9	19,411	22.0
Medicaid	5,644	3.5	1,877	9.1	1,440	6.6	529	1.7	1,798	2.0
Medicare	89,820	55.7	7,349	35.8	7,536	34.6	23,553	76.3	51,382	58.4
Defense/Military/Veteran	2,385	1.5	341	1.7	290	1.3	233	0.8	1,521	1.7
Indian/Public	220	0.1	65	0.3	54	0.2	31	0.1	70	0.1
Insurance, NOS	10,491	6.5	1,232	6.0	1,210	5.6	1,040	3.4	7,009	8.0
Unknown	16,840	10.4	4,453	21.7	6,618	30.4	1,081	3.5	4,688	5.3
Urban Rural by zip code										
Urban	150,025	93.1	18,998	92.5	20,259	93.0	28,966	93.8	81,802	92.9
Rural	11,203	6.9	1,530	7.5	1,530	7.0	1,900	6.2	6,243	7.1
AAMC 2005 teaching hospital										
Non-teaching hospital	149,258	92.6	18,574	90.5	20,184	92.6	29,165	94.5	81,335	92.4
Teaching hospital	11,970	7.4	1,954	9.5	1,605	7.4	1,701	5.5	6,710	7.6
Hospital volume										
Low	103,348	64.1	11,804	57.5	11,038	50.7	21,685	70.3	58,821	66.8
High	57,880	35.9	8,724	42.5	10,751	49.3	9,181	29.7	29,224	33.2

SES = Socioeconomic Status (percent living below poverty line); Lowest (≥20%); Middle-low (≥10% and <20%); Middle-high (≥5% and <10%); Highest (<5%).

**Table 2:** **Pathological and clinical characteristics**

Variable		All patients	Marital status at DX							
				Never married		Separated/Divorced		Widowed		Married
	N	%	N	%	N	%	N	%	N	%
All	161,228	100.0	20,528	100.0	21,789	100.0	30,866	100.0	88,045	100.0
Co-morbidity										
None	12,754	7.9	2,978	14.5	3,509	16.1	1,516	4.9	4,751	5.4
1 ~ 2	3,793	2.4	667	3.2	761	3.5	583	1.9	1,782	2.0
3 ~ 4	8,477	5.3	1,216	5.9	1,348	6.2	1,544	5.0	4,369	5.0
>4	136,204	84.5	15,667	76.3	16,171	74.2	27,223	88.2	77,143	87.6
SEER stage										
Localized	26,672	16.5	2,316	11.3	2,572	11.8	5,632	18.2	16,152	18.3
Regional, direct extension ± lymph nodes	19,478	12.1	2,184	10.6	2,153	9.9	3,765	12.2	11,376	12.9
Regional, lymph nodes only	13,820	8.6	1,371	6.7	1,486	6.8	2,697	8.7	8,266	9.4
Distant	64,374	39.9	8,049	39.2	7,415	34.0	12,571	40.7	36,339	41.3
Unknown/Unstaged	36,884	22.9	6,608	32.2	8,163	37.5	6,201	20.1	15,912	18.1
Types of lung cancer										
SCLC	20,073	12.5	2,250	11.0	2,358	10.8	4,012	13.0	11,453	13.0
NSCLC	96,134	59.6	10,493	51.1	10,270	47.1	18,589	60.2	56,782	64.5
Other	45,021	27.9	7,785	37.9	9,161	42.0	8,265	26.8	19,810	22.5
Grade										
Undifferentiated	11,780	7.3	1,264	6.2	1,399	6.4	2,292	7.4	6,825	7.8
Poorly-differentiated	37,134	23.0	4,161	20.3	4,049	18.6	6,745	21.9	22,179	25.2
Moderately-differentiated	18,492	11.5	1,808	8.8	1,897	8.7	3,492	11.3	11,295	12.8
Well-differentiated	5,654	3.5	507	2.5	535	2.5	1,188	3.8	3,424	3.9
Unknown/not stated	88,168	54.7	12,788	62.3	13,909	63.8	17,149	55.6	44,322	50.3
Regional nodes positive										
No	19,699	12.2	1,737	8.5	2,066	9.5	3,358	10.9	12,538	14.2
Yes	11,604	7.2	1,105	5.4	1,271	5.8	1,770	5.7	7,458	8.5
Unknown	129,925	80.6	17,686	86.2	18,452	84.7	25,738	83.4	68,049	77.3
Chemotherapy										
No	93,242	57.8	10,371	50.5	9,716	44.6	22,128	71.7	51,027	58.0
Yes	51,037	31.7	5,855	28.5	5,933	27.2	7,395	24.0	31,854	36.2
Unknown	16,949	10.5	4,302	21.0	6,140	28.2	1,343	4.4	5,164	5.9
Radiation Therapy										
No	46,765	29.0	5,948	29.0	5,054	23.2	12,691	41.1	23,072	26.2
Yes	102,232	63.4	10,955	53.4	11,154	51.2	17,615	57.1	62,508	71.0
Unknown	12,231	7.6	3,625	17.7	5,581	25.6	560	1.8	2,465	2.8
Surgery										
No	114,045	70.7	13,607	66.3	12,571	57.7	24,659	79.9	63,208	71.8
Yes	34,896	21.6	3,144	15.3	3,534	16.2	5,794	18.8	22,424	25.5
Unknown	12,287	7.6	3,777	18.4	5,684	26.1	413	1.3	2,413	2.7

SES = Socioeconomic Status (percent living below poverty line); Lowest (≥20%); Middle-low (≥10% and <20%); Middle-high (≥5% and <10%); Highest (<5%).

### Survival

**Figure 1:**
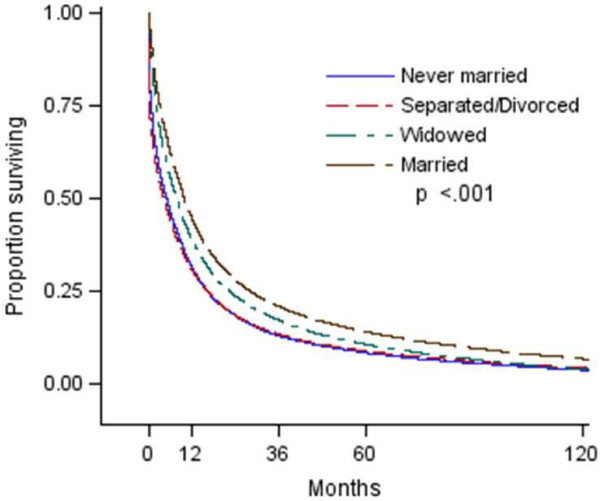
**This figure illustrates proportion surviving by marital status.**

Median survival time (MST) in months and survival rates at 1-, 3-, and 5-years post-diagnosis are displayed in Table 3 and Figure 1. Married patients had the longest MST (9.9 months), followed by widowed patients (7.7 months), while never separated/divorced patients had the shortest (4.1 months). The 1-year survival rate was longest for married (44.5%) and widowed (38.8%) patients, and markedly shortest for never married (31.5% and separated/divorced patients (30.6%). This pattern held for 3- and 5-year survival rates.

**Table 3:** **Median survival time and survival rates, n = 161,228**

	Median survival (months)	Survival rates (%) at time (yrs) after diagnosis		
		1 yr	3 yrs	5 yrs
Overall	8.1	39.9	18.2	12.1
Marital status				
Never married	4.9	31.5	13.0	8.4
Separated/Divorced	4.1	30.6	13.5	8.9
Widowed	7.7	38.8	17.2	10.7
Married	9.9	44.5	20.9	14.2
Race				
White	8.1	40.1	18.5	12.3
Black	7.0	36.2	14.5	8.9
Other	10.2	46.2	20.1	12.5
Hispanic origin				
No	8.0	39.8	18.2	12.1
Yes	8.4	40.5	17.9	12.1
SES				
Lowest	6.5	34.8	13.8	8.7
Middle-Low	7.6	38.3	16.8	11.0
Middle-High	8.5	41.1	19.4	12.8
Highest	9.5	44.0	21.7	15.1

SES = Socioeconomic Status (percent living below poverty line); Lowest (≥20%); Middle-low (≥10% and <20%); Middle-high (≥5% and <10%); Highest (<5%).

### Regression analysis

Results from the 5 Cox proportional hazards regression models are shown in Table 4. In the univariate model, compared to never married, a protective effect was found for married (HR 0.70; 95% CI = 0.69-0.71) and widowed (HR 0.81; 95% CI = 0.80-0.83) patients, while separated/divorced patients had slightly worse survival (HR 1.03; 95% CI = 1.01-1.05). When the final model was adjusted for all covariates (model 5), being married (HR 0.85; 95% CI = 0.81-0.89) and widowed (HR 0.88; 95% CI = 0.84-0.93) remained positively associated with better survival compared with never married, and the detrimental association of separated/divorced (HR 1.05; 95% CI = 1.02-1.08) with survival remained.

**Table 4:** **Proportional cox regression models, n = 161,228**

		Model 1		Model 2		Model 3		Model 4		Model 5	
Prognostic factors	Category	HR (95% CI)	P value	HR (95% CI)	P value	HR (95% CI)	P value	HR (95% CI)	P value	HR (95% CI)	P value
Marital status	Never married	1.00		1.00		1.00		1.00		1.00	
	Separated/ Divorced	1.03 (1.01, 1.05)	0.003	1.03 (0.89, 1.20)	0.654	1.03 (0.96, 1.10)	0.461	1.04 (1.01, 1.07)	0.008	1.05 (1.02, 1.08)	<.001
	Widowed	0.81 (0.80, 0.83)	<.001	0.82 (0.59, 1.14)	0.240	0.77 (0.63, 0.94)	0.010	0.87 (0.82, 0.91)	<.001	0.88 (0.84, 0.93)	<.001
	Married	0.70 (0.69, 0.71)	<.001	0.71 (0.53, 0.95)	0.021	0.70 (0.60, 0.83)	<.001	0.82 (0.78, 0.87)	<.001	0.85 (0.81, 0.89)	<.001
Race	White	1.00		1.00		1.00		1.00		1.00	
	Black	1.12 (1.10, 1.14)	<.001	0.97 (0.88, 1.05)	0.438	1.04 (1.01, 1.07)	0.021	0.99 (0.95, 1.02)	0.472	0.99 (0.95, 1.02)	0.391
	Other	0.91 (0.85, 0.97)	0.005	0.91 (0.85, 0.97)	0.007	1.00 (0.93, 1.08)	0.944	0.96 (0.89, 1.04)	0.314	0.85 (0.78, 0.93)	<.001
Hispanic origin	Non-Hispanic	1.00		1.00		1.00		1.00		1.00	
	Hispanic	0.98 (0.96, 1.01)	0.148	0.93 (0.85, 1.02)	0.130	0.97 (0.90, 1.06)	0.499	0.94 (0.89, 0.99)	0.026	0.91 (0.86, 0.96)	<.001
SES	Lowest	1.00		1.00		1.00		1.00		1.00	
	Middle-Low	0.90 (0.89, 0.92)	<.001	0.93 (0.90, 0.97)	<.001	0.95 (0.92, 0.98)	<.001	0.96 (0.93, 0.98)	0.002	0.96 (0.94, 0.99)	0.005
	Middle-High	0.84 (0.83, 0.85)	<.001	0.88 (0.84, 0.93)	<.001	0.90 (0.86, 0.93)	<.001	0.92 (0.89, 0.95)	<.001	0.92 (0.90, 0.95)	<.001
	Highest	0.77 (0.76, 0.79)	<.001	0.82 (0.77, 0.88)	<.001	0.85 (0.80, 0.91)	<.001	0.88 (0.85, 0.92)	<.001	0.89 (0.85, 0.92)	<.001

Model 1: Univariate.

Model 2: Multivariate only with Marital status + Race/Ethnicity/SES.

Model 3: Multivariate - Marital status + Race/Ethnicity/SES + demographics.

Model 4: Multivariate - Marital status + Race/Ethnicity/SES + demographics + clinical.

Model 5: Multivariate - Marital status + Race/Ethnicity/SES + demographics + clinical + individual comorbidities.

Notes: there is no interaction between marital status and race, ethnicity and SES respectively.

SES = Socioeconomic Status (percent living below poverty line); Lowest (≥20%); Middle-low (≥10% and <20%); Middle-high (≥5% and <10%); Highest (<5%).

## Discussion

Previous research has shown an association between marital status and survival in lung cancer, and that this association may be increasing over time (Kravdal & Syse 2011). For example, California Cancer Registry data has been used to test for overall associations of survival with marital status in lung cancer patients. This research found that for both extensive stage SCLC (HR 1.179; p < 0.001) and NSCLC (HR 1.175; 95% CI = 1.122-1.229), there are significant survival differences between unmarried and married patients (Ou et al. 2008; Ou et al. 2009). However, there are inconsistencies in the results of studies that have explored the relative survival disadvantage of different unmarried status categories. In addition, not all studies have been able to control well for treatment and comorbidity confounding variables. Thus, the goal of this study was to explore the association of marital status with survival following a diagnosis of lung cancer using data that is representative of the Florida state population and which allows for controlling for all demographic, clinical and comorbid variables. Our main finding was that married and widowed Floridian patients with lung cancer have a survival benefit compared with those who had never married, and that separated/divorced patients had worse survival than never married patients. These findings remained significant after inclusion of all demographic, clinico-pathologic, treatment and comorbidity variables in a fully adjusted Cox regression model.

Our findings are in concordance with some, but not all of the previous literature. Similar to our findings, Manzoli et al. (Manzoli et al. 2007) found that separated/divorced cancer patients had the worst survival of any marital status group. Conversely, a number of other study have found that never-married patients have worse survival than both widowed and separated/divorced patients (Pinquart & Duberstein 2010; Kravdal & Syse 2011; Kravdal 2013; Kravdal 2001), at least for some categories of patients. Early data from Norway (women diagnosed with cancer between 1996 and 1990 (Kvikstad et al. 1995)) showed that divorced women had an overall increased hazard ratio of 1.17 (95% CI = 1.07-1.27) for cancers including lung cancer compared to married women, whereas widows had no increased risk. However, in 2001, Kravdal (Kravdal 2001) found that for Norwegian women with lung cancer, being widowed was associated with the worst survival outcomes (HR 1.19; 95% CI = 1.09-1.30) compared with married women. The same study showed that, for male lung cancer patients, never married status was associated with the worst outcomes (HR 1.23; 95% CI = 1.16-1.30), whereas widowhood was associated with only half that detrimental effect (HR 1.12; 95% CI = 1.10-1.20). In the most recent data from Norway, a status of never married was found to be worst for both men and women with lung cancer, but the order of the relationship of widowed and divorced/separated status to survival was different for men and women (Kravdal 2013).

Other studies have divided divorced and separated individuals into discrete categories. One such study found that separated status carried the worst survival outcomes for 5-year and 10-year relative survival for cancer patients – approximately 72% and 64% the survival time of married patients (Sprehn et al. 2009). Another study (Lai et al. 1999), which explored SEER data for each cancer type separately, found the relative risk scores (compared to married) to be 1.18 for single, 1.16 for separated, 1.13 for divorced, and 1.08 for widowed male lung cancer patients (all significant differences); but no significant difference among relative risk scores for females.

Although many studies have found differences, albeit in inconsistent ways, among the different categories of unmarried individuals, this is not true for across the board. A review of the effect of marriage on survival broadly (Rendall et al. 2011) found little or no differences between never married, separated/divorced, and widowed statuses. A study of lung cancer in Japan found no significant increased risk of death in widowed female lung cancer patients compared to married patients, and no significant increased risk of death for separated/divorced male or female patients compared to married patients although widowed males patients had increased risk of death (HR 1.7; 95% CI = 1.2-2.5) (Saito-Nakaya et al. 2008).

One way that our results differ from much of the previous findings in the literature e.g., (Kravdal & Syse 2011; Saito-Nakaya et al. 2008; Kravdal 2013; Lai et al. 1999) is that we did not find differences between men and women in the relationship between marital status and survival. As gender and marital status interaction term in our Cox regression was not significant, indicating that marital status has the same modifying effect on survival in both genders, although gender does have a significant direct effect on survival, with males having worse survival then females with lung cancer (results not shown). The reason for this difference in our population from previous findings is unclear.

Our findings and these others suggest that some aspect of marriage and social networks in general seem to afford patients a comparatively longer time before succumbing to a disease. Previous studies on marriage and survival focused on the social support benefits that married couples have compared with never married or divorced/separated. For example, Pinquart (Pinquart & Duberstein 2010) posited that social networks, which would include marriage, would have effects on: biological pathways (neuroendocrine or neuro-immune pathways), health behaviors, access to health care systems and assistance with navigating its complexities, the likelihood of receiving vigorous and aggressive, active cancer treatment, and psychological consequences. All of these could have direct and/or indirect effects on survival. Empirically, Luszczynska, et al. (Luszczynska et al. 2012) found that patients with perceived/received family support had improved psychological and physical quality of life. Stress-related psychosocial factors have been shown to have a deleterious effect on survival in patients with lung cancer (Chida et al. 2008). Taniguchi et al. (Taniguchi et al. 2003) found that men who were not married had more psychological distress than married men (Umberson 1992). Lastly, married couples have been shown to engage in healthier lifestyle behaviors and less risky behaviors compared with unmarried couples (Krieger 1992).

This study had some limitations. It was a cross-sectional study so causality could not be assessed. However, as this was a linkage of databases some of the information was collected at a later time period. The databases that we have access to do not have individual-level indicators of SES; therefore, we used neighborhood-level poverty as a proxy. However, using neighborhood indicators of SES has been shown to be a valid and reliable methodology (29). Also, marital status was determined only at the time of diagnosis and patients’ status may have changed over time.

Our study showing marital status is a strong independent predictor of survival was unique in that we had a linkage of two large databases: 1) the FCDS registry containing incident cancer cases plus other demographic information and 2) AHCA database, providing codes for diagnoses and procedures received as the patient went forward with treatments for a large age range of patients (18–110 years). In addition, we had valid proxy of individual SES information utilizing information from the U.S. Census. With this information we were able to control for demographic and clinico-pathological characteristics, (i.e., tumor characteristics, hospital type, treatments) as well as comprehensive comorbidities.

## Conclusions

We found strong evidence that married and widowed patients with lung cancer fare better in terms of survival than those who never married even after adjusting for some extensive factors including some associated with social support, whereas divorced/separated patients did worse. This suggests that some other factor(s) associated with marriage – even after the marriage has ended through widowhood, but not divorce or separation– are associated with survival. Further research to fully understand these factors and how the beneficial effect can be extended to those who have never been married or have had marriage terminated through separation or divorce is needed.
